# Making shared decisions with older men selecting treatment for lower urinary tract symptoms secondary to benign prostatic hyperplasia (LUTS/BPH): a pilot randomized trial

**DOI:** 10.1186/s41687-022-00519-x

**Published:** 2022-10-15

**Authors:** Haidee Ngu, Shu Hui Neo, Eileen Yi Leng Koh, Henry Ho, Ngiap Chuan Tan

**Affiliations:** 1 c/o SingHealth Polyclinics, 167, Jalan Bukit Merah, Connection One, Tower 5, #15-10, 150167 Singapore, Singapore; 2 Singapore General Hospital, Singapore, Singapore; 3 SingHealth-Duke NUS Family Medicine Academic Clinical Programme, Singapore, Singapore

**Keywords:** Older men, Benign prostatic hyperplasia, Physician training, Shared decision-making, Symptom score

## Abstract

**Background:**

Making high-quality decisions when selecting treatment for lower urinary tract symptoms due to benign prostatic hyperplasia (LUTS/BPH) requires a shared decision-making approach. However, older people with lower health literacy face barriers. The pilot study aimed to evaluate the feasibility of recruiting participants and evaluate the effectiveness of a multi-level intervention on decision quality for the treatment of LUTS/BPH.

**Method:**

In this 2-arm, randomized controlled trial, multi-ethnic Asian men aged ≥ 50 years with moderate or severe symptoms (IPSS ≥ 8 and/or QOL ≥ 3) and physicians were recruited at a Singapore public primary care clinic. Men were randomized to either physicians trained in shared decision-making and used a pictorial patient-reported symptom score (Visual Analogue Uroflowmetry Score) during the consultation or to physicians untrained in shared decision-making who did not use the score. Decision quality was measured using SDMQ-9 scores from men and their physicians after the consultation.

**Results:**

60 men (intervention [n = 30], control [n = 30]) receiving care from 22 physicians were recruited. Men’s mean age was 70 ± 9 years: 87% were Chinese, 40% had no formal education, and 32% were of lower socioeconomic status. No difference in decision quality from the men’s nor their physicians’ perspectives was noted [for men: mean score = 70.8 (SD 20.3) vs. 59.5 (SD 22.4); adjusted p = 0.352] [for physicians: mean score = 78.1 (SD 14.1) vs. 73.2 (SD 19.8); adjusted p > 0.999].

**Conclusion:**

It was feasible to recruit the intended participants. There was no difference in decision quality between men who used shared decision-making and usual care for the treatment of LUTS/BPH.

**Supplementary Information:**

The online version contains supplementary material available at 10.1186/s41687-022-00519-x.

## Introduction

Lower urinary tract symptoms secondary to benign prostatic hyperplasia (LUTS/BPH) is a common condition among older men. Its incidence increases with rising age. [1] Worldwide, the prevalence of LUTS ranges from 16.5 to 62.8% and is set to further increase because of aging populations. [2, 3, 4] If untreated, LUTS/BPH can cause complications such as acute or chronic urinary retention, urinary tract infection, renal dysfunction, incontinence, and erectile dysfunction. [5].

Treatment decisions can be complex, involving significant trade-offs and decisional conflict. [6] The risk-benefit ratio depends on age, symptom severity, health status, and other factors. Treatment often improves symptoms [7], but not all individuals benefit from treatment. [8] The type of treatment chosen (watchful waiting, medications, surgery) depends on individual preferences. This requires a style of consultation known as shared decision-making (SDM). It has been defined by Elwyn as “an approach where physicians and patients share the best available evidence when faced with the task of making decisions, and where patients are supported to consider options, to achieve informed preferences.” [9] Studies have shown that when shared decision-making is used, people make high-quality decisions, have greater satisfaction, better medication adherence, and improved clinical outcomes. [10, 11].

Barriers to shared decision-making include the limited teaching of this skill in under- and postgraduate medical training [12] and physicians’ assumption that shared decision-making takes up too much time or is unsuitable for some patients. [13] Although older people prefer involvement in medical decision-making, [14], [15] there remains in some cultures, a prevailing paternalistic style of consultation where society is used to the mantra: ‘doctor knows best’. [16] Many older people are disadvantaged by lower literacy levels, cognitive, hearing, and visual impairment. 17 Decisions are further complicated by multimorbidity, drug-drug interactions, drug-disease interactions, and psychosocial circumstances. [18].

Shared decision-making can be facilitated by communication tools such as a patient decision aid. These tools are designed to present evidence in a clear and concise format to aid decision-making. [19] Studies have shown that pictograms used in decision aids can minimize complexity for older people with less education. [20, 21] However, current tools designed for LUTS/BPH cater to educated, English-speaking men. [22, 23, 24, 25] Some are web-based tools, rendering them inaccessible to older people with low health literacy who are less likely to be digitally savvy. [23, 24].

Recognizing the limitations faced by older people, the greatest scope for improving SDM may be within the area of physician training. Studies have shown that interventions to train healthcare professionals can improve their adoption of shared decision-making. [26, 27] Since physicians are the most trusted source of information for older people, the physician’s attitude and skill are pivotal in determining the extent to which the older person is involved in the decision-making process. [28] There has been a proliferation of physician training programs in recent years, including in Asian countries such as South Korea and Japan. [29] Locally, the use of SDM to make high-quality decisions with patients is also gaining traction due to changing patient expectations, an increase in chronic diseases, and increased patient engagement. Primary care physicians are the first port of call for many older people who decide to seek treatment for their symptoms. Although it is common for them to care for people with LUTS/BPH, physician training in shared decision-making for this disease remains unexplored.

**Fig. 1:**
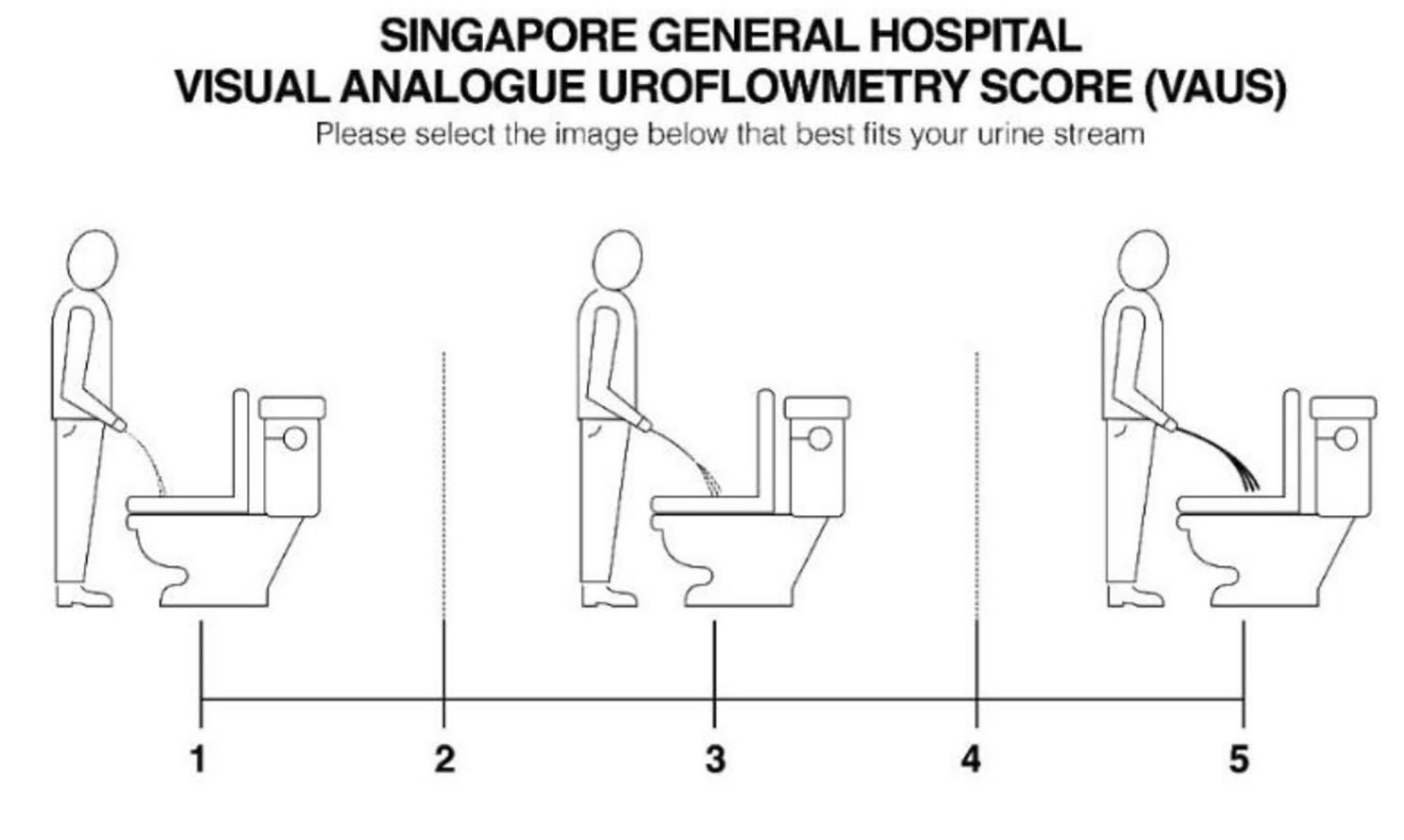
Visual analogue uroflowmetry score. A novel Likert score with 1 being slowest stream and least volume, and 5 being fastest stream and highest volume Raj Tiwari, Ng MY, Neo SH, R Mangat, H Ho. Prospective validation of a novel visual analogue uroflowmetry score (VAUS) in 1000 men with lower urinary tract symptoms (LUTS). World Journal of Urology 2020; 38(5);1267–1273 doi: 10.1007/s00345-019-02909-1

Training physicians to use a simple communication tool to support SDM has the potential to reach older people with lower literacy levels so that they too can make high-quality decisions about their treatment. Our intervention consisted of (1) training physicians in shared decision-making about treatment for LUTS/BPH in the primary care setting (2) a novel patient-reported symptom score (the Visual Analogue Uroflowmetry Score, VAUS, see Fig. 1) to support the shared decision-making consultation. The VAUS consists of a low-tech paper-based pictogram with an accompanying Likert scale to measure urine flow. It was developed and validated in a cohort of older, multi-ethnic Asian men in a urology clinic, the results of which have been published elsewhere. [30] The VAUS was designed to be elder-friendly and could be self-administered by people with lower literacy levels. In Asia, many men find the International Prostate Symptom Score (IPSS) tool too difficult to understand, particularly those with less education. [31] Language barriers for men who do not use English as a first language make interpretation difficult without validated translations. Although this pictogram is not a patient decision aid in the traditional sense, it may serve as a starting point toward a more person-centered consultation. By starting with the individual’s perspective using the VAUS, the SDM-trained physician can guide upfront goal-setting and share the potential treatment options that would best serve the individual’s goal.

The primary aim of this study was to pilot the feasibility of recruiting older Asian men with lower urinary tract symptoms and physicians in the primary care setting. The secondary aim was to evaluate the effect of a multi-level intervention (shared decision-making training and VAUS) compared to usual care (no shared decision-making training and no VAUS) on the decision quality of older Asian men seeking treatment for moderate or severe lower urinary tract symptoms. Dyadic evaluation of the decision quality was made using the patient-reported Shared Decision-Making Questionnaire (SDMQ-9) and the physician-reported Shared Decision-Making Questionnaire (SDMQ-Doc).

## Methods

### Study design

**Fig. 2:**
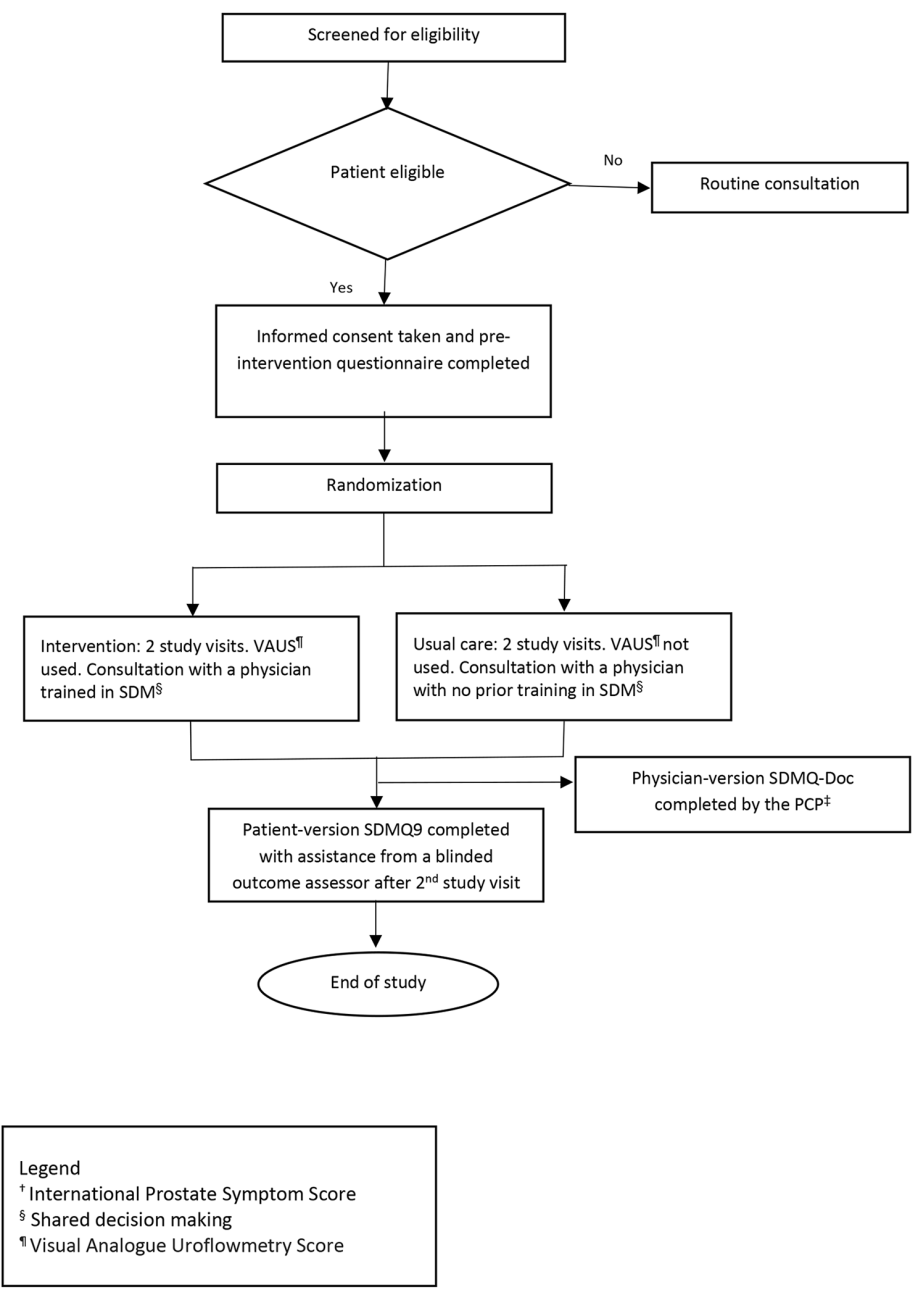
Flowchart of the study protocol

This study was a two-arm pilot randomized controlled trial. It comprised two study visits conducted two weeks apart (see Fig. 2). The first visit was to conduct a focused history, physical examination, and laboratory tests (urinalysis and prostate-specific antigen). The second visit was to confirm the diagnosis of LUTS/BPH and discuss treatment options. Due to the nature of the intervention, only outcome assessors were blinded.

### Setting

This study was conducted in a public primary care clinic (polyclinic) in Singapore, located in a housing estate in the southern region of the island state. The polyclinic premise occupies a single floor in a multi-story building. Physician offices are located in two clusters separated by a public corridor. The polyclinic provides a comprehensive range of primary care services, on-site pharmacy as well as allied health services. To ensure accessibility and affordability to these services, patients, particularly the lower to middle income and older individuals receive significant government subsidies. Hence, they constitute a large proportion of patient daily attendances. [32].

During the study, the polyclinic had 22 primary care physicians. The physicians on duty each day managed about 600 to 700 patients during office hours, of which about 60% were those for chronic disease management. [33] The housing area surrounding the polyclinic had approximately 151, 980 residents – a multi-ethnic Asian population of which 43% were older than 50 years of age, 48% were males, and comprised Chinese (79%), Indians (10%), Malays (8%) and other ethnic groups (3%). [34]

### Preparatory work for the study

Pre-testing of the SDMQ-9 questionnaire.

This questionnaire was selected for its short length, ease of administration, good internal consistency, and construct validity. [35, 36] Translated versions are available in the three main local languages; English, Mandarin, and Malay. [37] In this study, content validity was established by two primary care physicians who are experts in the field of shared decision-making. [38] It was then piloted on a convenience sample of five men already on treatment for LUTS/BPH but who were not enrolled in the study. Two men used the English version, two used the Mandarin version, and one used the Malay version. Completion rates of the questionnaire were 95% for all items indicating high acceptability. The average time taken to complete the questionnaire was eight minutes which all men found acceptable.

### Participants and recruitment

All polyclinic physicians were eligible physician-participants. Amongst patients, men aged 50 years and older attending the polyclinic for routine chronic disease management were conveniently sampled. They were eligible if they had an International Prostate Symptom Score (IPSS) of eight or more, indicating moderate or severe symptoms, and/or the overall Quality of Life score of three or more, indicating dissatisfaction due to symptoms. They could be of any local Asian ethnicity and could communicate in either of the three local languages.

Men were excluded if they had indwelling catheters, urinary incontinence, anuria, gross haematuria, acute urinary retention, urinary tract infection, and prostate cancer. Men receiving treatment for lower urinary tract symptoms or palliative care, had any disability including visual or hearing impairment which rendered them incapable of understanding the study procedure, and who had explicitly stated disinterest to the study team were also excluded.

The research assistant screened men for eligibility which was confirmed by the Principal Investigator. Reasons for ineligibility and reasons for non-recruitment where eligible was recorded in the study screening log. When recruitment faltered, the Principal Investigator reviewed the screening log to identify barriers and to introduce solutions to improve recruitment.

The patient information sheet and consent form were in English but administered in the older men’s preferred language. In the informed consent process, the research assistant emphasized the importance of completing the study. Upon obtaining written consent after their queries were addressed, the research assistant interviewed them using the pre-intervention questionnaire to collect sociodemographic data. Data on their comorbidities were obtained from electronic medical records. To reduce the frequency of drop-outs, phone calls were used to remind men to attend the follow-up study visit.

### Randomization

Eligible men who consented to the study were randomly assigned to physicians in either the intervention or control group in a 1:1 ratio according to a pre-defined computer-generated sequence. The study used 15 blocks of 4 patients with 2 in the intervention arm and 2 in the control by randomly selecting one of 6 possible permutations of the treatment among 4 patients in each block; AABB, ABAB, ABBA, BAAB, BABA, BBAA. The random selection of blocks was done using a list of random numbers generated using Excel. This predefined sequence of numbers was concealed in sequentially-numbered, sealed, opaque envelopes by a volunteer who was not involved in the study. Envelopes were opened after eligibility had been confirmed and written informed consent obtained.

### Treatment group: intervention

The intervention consisted of (1) a Continuing Medical Education-accredited shared decision-making training program for physicians (2) the VAUS tool used by trained physicians during the consultation.

The physicians attended two face-to-face training sessions conducted by two urologists (TR and NSH) and the Principal Investigator, a primary care physician (HN). Upon completion, they were awarded Continuing Medical Education credits by the Singapore Medical Council.

The first training session aimed to introduce the use of VAUS as a validated, elder-friendly tool to evaluate the severity of men with LUTS/BPH. The VAUS is a pictogram comprising three images with an accompanying Likert score of 1, 3, and 5 lined up with them. 2 and 4 are in between the images. The VAUS incorporates the flow rate (distance from patient to where the urine stream lands) and voided volume (thickness of the stream). Physicians could ask men to select any number from 1 to 5, with 1 being the slowest stream. In the univariate analysis reported in the earlier validation study, VAUS matched uroflowmetry voided volume and severe LUTS category IPSS for predicting poor flow (p < 0.001). Based on receiver operating characteristic curve analysis, the best cut-off for measuring poor flow was 2.5. [30].

The second session of the training covered the nine elements in the SDMQ-9 questionnaire. Common pitfalls in communication with older people of lower literacy were also discussed. SDM competencies were demonstrated using the SDM Magic training video clips. [39] After the training, physicians conducted a consultation with a simulated patient following which they received individual feedback and advice.

At the first study visit, men assigned to physicians in the intervention group were given the VAUS tool and instructed to complete it during the waiting time. They were instructed to submit it to their physician at the beginning of the consultation. To minimize disruption to the clinic workflow, the research assistant communicated with the clinical operations team to enable fast-paced adjustments to the appointment scheduling system as soon as the men were randomized. By doing so, the time allocated for each study visit was doubled to twenty minutes. The management of other medical issues was at the discretion of the physicians and according to local clinical practice. Consultations were carried out in the men’s preferred language using a translator if required. To encourage intervention fidelity, the Principal Investigator sent out email reminders regarding the study protocol and the elements of a shared decision-making consultation.

### Control group: usual care

Men assigned to physicians in the control group were instructed to raise their symptoms at the beginning of the twenty minutes consultation. VAUS was not provided. Although physicians were aware of the study visits, no attempt was made to standardize usual care.

### Outcomes

The feasibility outcomes were decided a-priori and included: (1) a recruitment rate of 10 men per month in the first two months rising to 20 men per month in the subsequent two months (2) the proportion of physicians participating in the pilot study would be 80% or greater (3) the drop-out rate would be < 10% and (3) the proportion of participants with missing data would be < 5%.

The secondary outcomes were the patient-reported SDMQ-9 and the physician reported SDMQ-Doc scores. The SDMQ-9 instrument consists of nine statements scored from 0 to 5 on a six-point Likert scale ranging from 0 (″*completely disagree*″) to 5 (″*completely agree*″). Standard scoring for the full scale was the composite score, from 0 to 45. Following the developer’s recommendations, the composite raw score was converted to a score out of 100. 100 reflected the highest possible level of shared decision-making. Higher scores indicated higher quality decisions. [36].

### Data collection

Immediately after the second study visit, men completed the patient-reported questionnaire. They were assisted by a blinded outcome assessor in a quiet room located outside the physicians’ office. All men received one $20 voucher each upon completing the study. The physician independently completed the physician version at the same time. Study personnel underwent training by HN to standardize and ensure consistency in the administration of the patient-reported questionnaire. To minimize erroneous data entry and missing data, they were trained to use Research Electronic Data Capture (REDCap), a secure browser-based web application for developing, maintaining, and managing collected data. [40] Results were entered into the application at the end of each day.

### Sample size

The primary aim of the study was to find out the feasibility of using a multi-level intervention for decision making among older Asian men with moderate or severe lower urinary tract symptoms. Based on Browne, [41] a small sample size of 30 per arm is adequate for a pilot study. With 30 per arm, the study has 80% power to detect an effect size between 0.1 and 0.3, based on Whitehead et al. [42] With an anticipated 10% drop out rate, from prevalence estimates of 20%,^2^ assuming 50% of eligible men will decline participation, a total of at least 420 will be screened for their eligibility using LUTS. The final sample size to be recruited in the pilot will be 30 per arm (n = 60).

### Data analysis

Descriptive statistics were used for pilot outcomes. Baseline characteristics were reported as frequencies and percentages. The Charlson Comorbidity Index data were not normally distributed; median and interquartile ranges were presented. Statistical analysis was performed using SPSS (Statistical Package for Social Sciences) Statistics V.24.0. Differences between patient and physician-reported scores were compared using the independent *t*-test. p-values were adjusted for multiple testing using the Holm-Bonferroni correction method. [43] The formula to calculate Holm-Bonferroni is overall alpha (0.05) divided by (number of tests-rank + 1). A p-value of < 0.05 was considered significant. Data was analyzed as intention-to-treat.

## Results

### Participants and recruitment

**Fig. 3:**
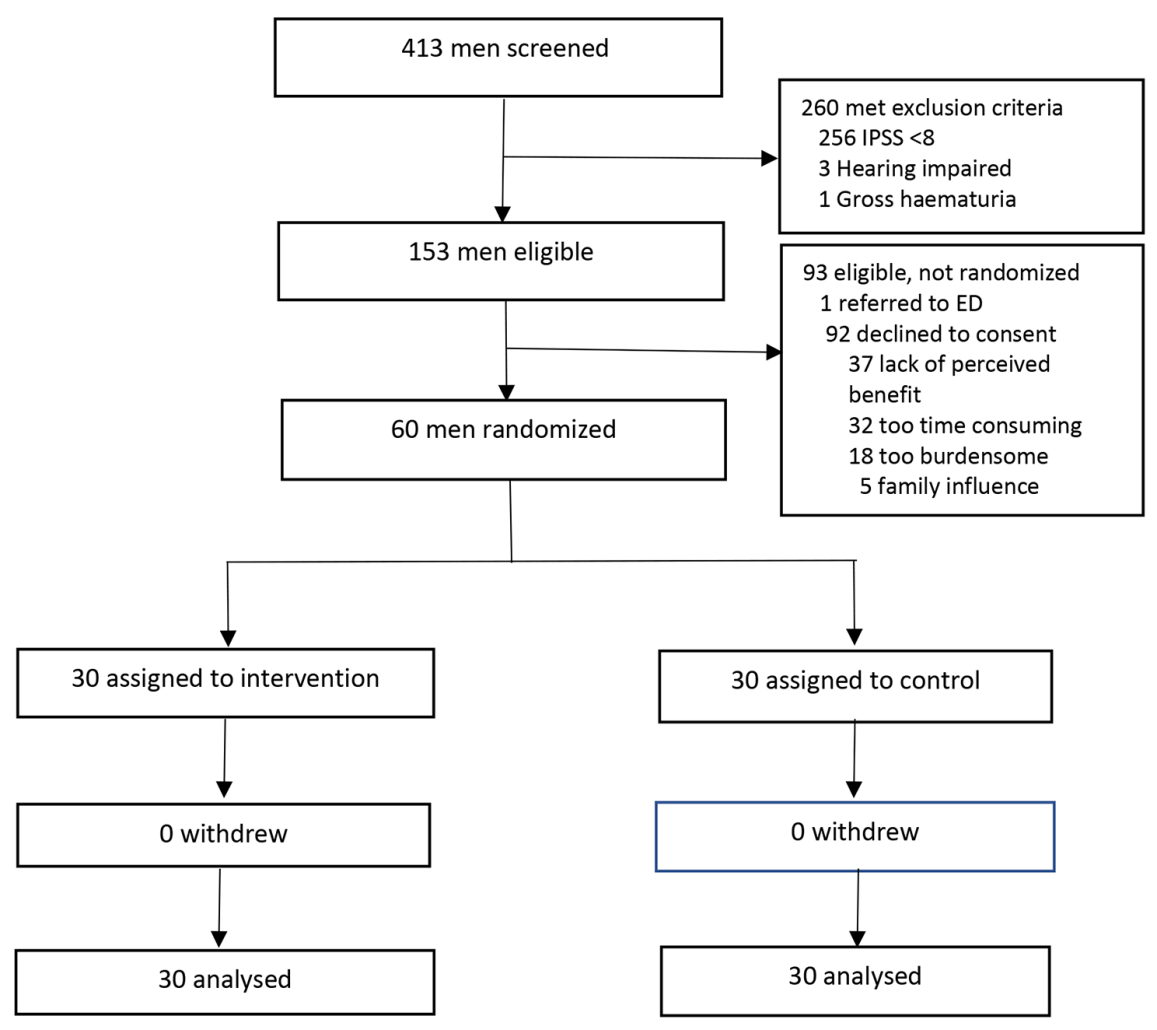
Patient flow diagram

Figure 3 shows the patient flow diagram. Of 413 men screened for eligibility, 153 were eligible and 60 men were randomized. Of the 260 ineligible men, 256 did not meet the IPSS cut-off of > 8, 3 were hearing impaired and 1 had gross haematuria. 60% of the eligible men (92/153) declined to participate. One was referred to the Emergency Department by his primary care physician for an unrelated complaint. Reasons for non-participation were the lack of perceived benefit (37/92, 40%), the lack of time (32/92, 35%), the burden of following the study procedure (18/92, 20%) and family influence (5/92, 5%). All men who underwent randomization completed the study. There were no drop-outs or missing data.

Table 1 describes baseline patient-level characteristics within the two groups. 87% of the men were Chinese, mean age was 70 ± 9 years; 40% of them had no formal education; 45% were unemployed or retired and 32% lived in public rental or small (1–2 room) apartments which was a proxy for lower socioeconomic status. 30.0% in the intervention group and 43% in the control group had severe symptoms (IPSS 20–35).

**Table 1:** Baseline characteristics of men by treatment group

Characteristics	Intervention, n (%)	Control, n (%)
	30 (50.0)	30 (50.0)
**Age**		
Below 65 years	11 (36.7)	10 (33.3)
65 years and above	19 (63.3)	20 (66.7)
**Ethnic Group**		
Chinese	25 (83.3)	27 (90.0)
Malay/ Indian/ Others	5 (16.7)	3 (10.0)
**Education**		
No formal education/ Primary	11 (36.7)	13 (43.3)
Secondary	13 (43.3)	9 (30.0)
Pre-U/ Diploma/University	6 (20.0)	8 (26.7)
**Employment status**		
Employed	14 (46.7)	19 (63.3)
Unemployed	7 (23.3)	4 (13.3)
Retired	9 (30.0)	7 (23.3)
**Housing Type**		
HDB 1–2 Room/ rental room/ Apartment	9 (30.0)	10 (33.3)
HDB 3–5 Room	20 (66.7)	18 (60.0)
Condo/ Landed	1 (3.3)	2 (6.7)
**Current Smoker**		
Yes	3 (10.0)	7 (23.3)
No	27 (90.0)	23 (76.7)
**IPSS**		
Moderate symptoms (8–19)	21 (70.0)	17 (56.7)
Severe symptoms (20–35)	9 (30.0)	13 (43.3)
**QoL**		
Dissatisfied (≥ 3)	29 (96.7)	30 (100.0)
Satisfied (< 3)	1 (3.3)	0 (0.0)
**Charlson Comorbidity Index**, Median (IQR)^1^	4 (4–5)	4 (4–5)

^1^IQR = inter-quartile range

All 22 eligible primary care physicians participated in the study. In terms of physician characteristics, the sample was mostly female, aged 40 years and above, and had been practicing for more than ten years. (Table 2).

**Table 2:** Baseline characteristics of primary care physicians by treatment group

Characteristics	Intervention, n (%)	Control, n (%)
	11 (50.0)	11 (50.0)
**Age**		
Below 40 years	4 (18.2)	6 (27.3)
40 years and above	7 (31.8)	5 (22.7)
**Sex**		
Male	3 (13.6)	2 ( 9.1)
Female	8 (36.4)	9 (40.9)
**Years since graduation**		
Below 10 years	2 ( 9.1)	4 (18.2)
10 years and above	9 (40.9)	7 (31.8)

The number of men recruited each month is shown in Table 3. The recruitment period was increased from four to six months to reach the target sample size. The overall eligibility fraction (proportion of potential participants who undergo screening and are eligible to enroll, 153/413) was 37%. The enrollment fraction (proportion of people who are eligible for participation and who enroll, 60/153) was 39%. The recruitment fraction (proportion of potential participants who enrolled was 60/413) was 15%. The number needed to screen to identify one person eligible for enrollment 1/recruitment fraction) was 7.

**Table 3:** Number of men recruited per month

	Month					
**Number of men**	**1**	**2**	**3**	**4**	**5**	**6**
Screened, n = 413	38	45	55	85	128	62
Eligible, n = 153	6	10	17	25	66	29
Randomized, n = 60	3	2	7	14	22	12

### SDMQ9 and SDMQ-Doc

Table 4 summarizes the patient-reported scores. Men in the intervention group reported higher composite scores compared to men in the control group however this did not reach statistical significance when adjusted for multiple comparisons [mean score = 70.8 (SD 20.3) vs. 59.5 (SD 22.4); adjusted p = 0.352]. While all individual 9 item-level means were higher in the intervention group, none were statistically significant.

**Table 4:** Comparison of SDMQ-9 scores among men in the intervention and control groups

	Intervention	Control (n = 30)	^a^p-value	^b^ Adjusted p-value (Holm-Bonferroni Method)
	**(N = 30)**			
**Total SDMQ-9 score, Mean (SD)**	70.8 (20.3)	59.5 (22.4)	0.044	0.352
1. My doctor made clear that a decision needs to be made	4.3 (0.9)	4.1 (1)	0.5	> 0.999
2. My doctor wanted to know exactly how I want to be involved in making the decision	3.1 (1.9)	2.7 (1.7)	0.426	> 0.999
3. My doctor told me that there are different options for treating his/her medical condition	4.0 (1.4)	2.9 (1.7)	0.007	0.07
4. My doctor precisely explained the advantages and disadvantages of the treatment options	2.4 (1.8)	2.1 (1.6)	0.496	> 0.999
5. My doctor helped me understand all the information	3.5 (1.4)	2.6 (1.6)	0.033	0.297
6. My doctor asked me which treatment option I prefer	3.6 (1.5)	2.9 (1.7)	0.119	0.714
7.My doctor and I thoroughly weighed the different treatment options	3.0 (1.6)	2.5 (1.7)	0.246	0.984
8. My doctor and I selected a treatment option together	3.9 (1.4)	3.3 (1.7)	0.133	0.714
9. My doctor and I reached an agreement on how to proceed	4.2 (1.1)	3.6 (1.5)	0.096	0.672

Individual items are scored from 0 to 5 on a six-point Likert scale ranging from 0 (″*completely disagree*″) to 5 (″*completely agree*″). The composite raw score was multiplied by twenty and divided by nine to obtain a new composite score that ranged from 0 to 100 where 100 reflected the highest possible level of shared decision-making

^a^ Refers to the observed p-value before adjustment

^b^ Refers to the adjusted p-value using Holm-Bonferroni Method

The difference in the physician-reported composite score was not significant [mean score = 78.1 (SD 14.1) vs. 73.2 (SD 19.8); adjusted p > 0.999] (see Table 5). Individual 9-item level means were higher in the intervention group but none reached statistical significance.

**Table 5:** Comparison of SDMQ-Doc scores among the physicians in the intervention and control groups

		Intervention	Control		^b^ Adjusted p-value (Holm-Bonferroni Method)
		**(N = 30)**	**(n = 30)**	^**a**^ **p-value**	
**Total SDMQ-Doc score, Mean (SD)**		78.1 (14.1)	73.2 (19.8)	0.268	> 0.999
1.I made clear to my patient that a decision needs to be made		4.1 (0.7)	3.9 (0.9)	0.27	> 0.999
2.I wanted to know exactly from my patient how he/she wants to be involved in making the decision		3.8 (0.9)	3.4 (1.2)	0.153	> 0.999
3.I told my patient that there are different options for treating his/her medical condition		4.0 (1)	3.9 (1.1)	0.715	> 0.999
4.I precisely explained the advantages and disadvantages of the treatment options to my patient		3.7 (1)	3.4 (1.3)	0.24	> 0.999
5.I helped my patient understand all the information		3.8 (0.9)	3.6 (1.3)	0.555	> 0.999
6.I asked my patient which treatment option he/she prefers		4.0 (1.1)	3.9 (1.2)	0.661	> 0.999
7.My patient and I thoroughly weighed the different treatment options		3.6 (0.9)	3.3 (1.3)	0.286	> 0.999
8.My patient and I selected a treatment option together		3.9 (1)	3.6 (1.2)	0.255	> 0.999
9.My patient and I reached an agreement on how to proceed		4.1 (0.7)	3.9 (0.8)	0.311	> 0.999

Individual items are scored from 0 to 5 on a six-point Likert scale ranging from 0 (″*completely disagree*″) to 5 (″*completely agree*″). The total raw score was multiplied by twenty and divided by nine to obtain a new composite score that ranged from 0 to 100 where 100 reflected the highest possible level of shared decision-making

^a^ Refers to the observed p-value before adjustment

^b^ Refers to the adjusted p-value using Holm-Bonferroni Method

## Discussion

To the best of our knowledge, this study is among the first to evaluate the feasibility of using shared decision-making to improve the decision quality of older multi-ethnic Asian men selecting treatment for LUTS/BPH. The results support the feasibility of conducting a full-scale randomized trial involving dyads of primary care physicians and patients. Although the recruitment of men posed challenges, the complete participation of physicians, absence of drop-outs and missing data suggest that the study protocol is acceptable and can be integrated into existing workflows. Although they did not attain levels of statistical significance, scores reflecting shared decision-making were higher in the intervention group compared to those in the control group. These findings challenge the common preconception that SDM may be inappropriate for older men with lower literacy.

Recognizing that recruitment of older adults can be complex and time-consuming, [44] the inclusion and exclusion criteria were liberalized, the intervention was planned to coincide with routine visits, men were reminded through telephone calls, and missed follow-up visits were rescheduled at the men’s convenience. The research assistant coordinated with the clinic operations staff to ensure minimal disruption to clinic workflow. The outcome measure was chosen for its relevance to men and its ease of administration. Special attention was paid to the social and cultural considerations affecting the recruitment and retention of the multi-racial population. Using a common language relieves the uncertainty people feel about sharing and receiving information. [45] The multi-racial research team was proficient in the three main local languages. Standardized translations of the IPSS and SDMQ-9 were used. Interviews and healthcare consultations were conducted in men’s preferred language.

Although the sample size was reached, recruitment was slower than projected due to staffing issues and time constraints. It improved after additional study personnel were deployed. The overall recruitment fraction was 15%. This was significantly lower than the median recruitment fraction of 54.0% (interquartile range, 32.0–77.1%) in 81 other randomized controlled trials. [46] While the median number of subjects screened in these studies was 1.8, we had to screen 7 men to recruit one participant. The literature on the willingness of older people to participate in research suggests that willingness to participate decreases with age. [47] In our study, four categories of patient-related barriers were identified. These will need to be addressed for the future trial. First, older men could have perceived that their symptoms were part of normal aging, hence they declined to participate even if the study protocol was acceptable to them. Second, the long waiting time in the polyclinic is inevitable due to the high patient attendances. Third, family members accompanying men to the clinic may have competing responsibilities and were unable to bring them for the follow-up visits. Trust borne out of a long-term relationship with a relevant, known and trusted stakeholder such as a community nurse will be vital to address specific concerns and rectify misconceptions of the trial.

Men in the intervention group reported higher quality decisions compared to their physicians. Although not significant after adjusting for multiplicity, the differing perspectives of men and physicians in the complex, dynamic process of decision-making warrant further investigation in a large, adequately powered trial. Other studies have used audio or direct observations. [48] Scholl and colleagues have found that observer-based instruments do not correlate significantly with patient-reported measures. [49] Observer assessments are also susceptible to the Hawthorne effect. which may potentially result in an inflated estimate of effect size. [50].

Strength and limitations.

The physician training program and the use of VAUS to support shared decision-making are the strengths of the study. They were designed for decision-making with older men with LUTS/BPH and targeted those with lower literacy. The high participation rate among the primary care physicians reflected their recognition of the relevance of shared decision-making in clinical practice. Despite convenience sampling, the ethnic distribution of men in this study population is correlated with that of the local Asian male population. Men were randomized with concealed and computer-generated allocation and outcome assessment was blinded to prevent bias.

This trial had some important limitations. The generalizability of the findings is limited because men were not randomly selected. Response bias remains possible as men might feel obliged to give socially acceptable answers in the questionnaires, even though they were accorded autonomy by the research assistant. Cross-contamination may have occurred because trained physicians could influence untrained colleagues. The sample size was insufficient to detect a difference in the effectiveness of the intervention. Finally, participants were not involved in developing this study protocol. With the recently formed SingHealth Polyclinics patient advocacy support group for research, men from the community will be involved in the design of the future trial.

### Future implications

The impact of interventions aimed at incorporating SDM into routine clinical care should be evaluated from the perspectives of the physician-patient dyad. Outcomes such as the magnitude of lower urinary tract symptom control and improvement in quality of life should form part of the outcome assessment. Physician-led primary care teams consisting of nurse specialists and pharmacists are increasingly used to provide continuing and comprehensive care to older people. An inter-disciplinary approach to SDM training can be scaled up, given the increasing complexity of managing older people with multiple co-morbidities.

## Conclusion

The results of the study suggest the feasibility of recruiting older men and physicians in the primary care setting. However, multifactorial barriers to timely recruitment need to be addressed. There were no significant differences in decision quality between men who used shared decision-making and usual care for the treatment of LUTS/BPH.

## Electronic supplementary material

Below is the link to the electronic supplementary material

Supplementary Material 1

Supplementary Material 2

## Data Availability

The datasets used during the current study are available from the corresponding author on reasonable request.

## List of abbreviations

IPSS: International Prostate Symptoms Score
LUTS/BPH: Lower urinary tract symptoms due to benign prostatic hypertrophy
PDA: Patient decision aid
QOL: Quality of life
SDM: Shared decision making
VAUS: Visual analogue uroflowmetry score
