# Supplementary material for: Making shared decisions with older men selecting treatment for lower urinary tract symptoms secondary to benign prostatic hyperplasia (LUTS/BPH): a pilot randomized trial
Source: J Patient Rep Outcomes. 2022 Oct 15;6:112. doi: 10.1186/s41687-022-00519-x (PMC9569273; doi:10.1186/s41687-022-00519-x)
Supplement: Supplementary file 1 — Supplementary Material 1 [file 41687_2022_519_MOESM1_ESM.docx]

**Tables**

Table 1. Baseline characteristics of men by treatment group

| **Characteristics** | **Intervention, n (%)** | **Control,**  **n (%)** |  |
| --- | --- | --- | --- |
|  | 30 (50.0) | 30 (50.0) |  |
| **Age** |  |  |  |
| Below 65 years | 11 (36.7) | 10 (33.3) |  |
| 65 years and above | 19 (63.3) | 20 (66.7) |  |
| **Ethnic Group** |  |  |  |
| Chinese | 25 (83.3) | 27 (90.0) |  |
| Malay/ Indian/ Others | 5 (16.7) | 3 (10.0) |  |
| **Education** |  |  |  |
| No formal education/ Primary | 11 (36.7) | 13 (43.3) |  |
| Secondary | 13 (43.3) | 9 (30.0) |  |
| Pre-U/ Diploma/University | 6 (20.0) | 8 (26.7) |  |
| **Employment status** |  |  |  |
| Employed | 14 (46.7) | 19 (63.3) |  |
| Unemployed | 7 (23.3) | 4 (13.3) |  |
| Retired | 9 (30.0) | 7 (23.3) |  |
| **Housing Type** |  |  |  |
| HDB 1-2 Room/ rental room/ Apartment | 9 (30.0) | 10 (33.3) |  |
| HDB 3-5 Room | 20 (66.7) | 18 (60.0) |  |
| Condo/ Landed | 1 (3.3) | 2 (6.7) |  |
| **Current Smoker** |  |  |  |
| Yes | 3 (10.0) | 7 (23.3) |  |
| No | 27 (90.0) | 23 (76.7) |  |
| **IPSS** |  |  |  |
| Moderate symptoms (8 - 19) | 21 (70.0) | 17 (56.7) |  |
| Severe symptoms (20 - 35) | 9 (30.0) | 13 (43.3) |  |
| **QoL** |  |  |  |
| Dissatisfied (≥3) | 29 (96.7) | 30 (100.0) |  |
| Satisfied (<3) | 1 (3.3) | 0 (0.0) |  |
| **Charlson Comorbidity Index,** Median (IQR)^1^ | 4 (4-5) | 4 (4-5) |  |

^1^ IQR = inter-quartile range

Table 2. Baseline characteristics of primary care physicians by treatment group

| **Characteristics** | **Intervention, n (%)** | **Control, n (%)** |
| --- | --- | --- |
|  | 11 (50.0) | 11 (50.0) |
| **Age** |  |  |
|  |  |  |
| Below 40 years | 4 (18.2) | 6 (27.3) |
|  |  |  |
| 40 years and above | 7 (31.8) | 5 (22.7) |
|  |  |  |
| **Sex** |  |  |
|  |  |  |
| Male | 3 (13.6) | 2 ( 9.1) |
|  |  |  |
| Female | 8 (36.4) | 9 (40.9) |
|  |  |  |
| **Years since graduation** |  |  |
|  |  |  |
| Below 10 years | 2 ( 9.1) | 4 (18.2) |
|  |  |  |
| 10 years and above | 9 (40.9) | 7 (31.8) |
|  |  |  |

Table 3. Number of men recruited per month

|  | **Month** | | | | | |
| --- | --- | --- | --- | --- | --- | --- |
| **Number of men** | **1** | **2** | **3** | **4** | **5** | **6** |
| Screened, n=413 | 38 | 45 | 55 | 85 | 128 | 62 |
|  |  |  |  |  |  |  |
| Eligible, n=153 | 6 | 10 | 17 | 25 | 66 | 29 |
|  |  |  |  |  |  |  |
| Randomized, n=60 | 3 | 2 | 7 | 14 | 22 | 12 |

Table 4. Comparison of SDMQ-9 scores among men in the intervention and control groups

|  | **Intervention** | **Control (n=30)** | **^a^p-value** | **^b^ Adjusted p-value (Holm-Bonferroni Method)** |
| --- | --- | --- | --- | --- |
|  | **(N=30)** |  |  |  |
| **Total SDMQ-9 score, Mean (SD)** | 70.8 (20.3) | 59.5 (22.4) | 0.044 | 0.352 |
| **1. My doctor made clear that a decision needs to be made** | 4.3 (0.9) | 4.1 (1) | 0.5 | >0.999 |
| **2. My doctor wanted to know exactly how I want to be involved in making the decision** | 3.1 (1.9) | 2.7 (1.7) | 0.426 | >0.999 |
| **3. My doctor told me that there are different options for treating his/her medical condition** | 4.0 (1.4) | 2.9 (1.7) | 0.007 | 0.07 |
| **4. My doctor precisely explained the advantages and disadvantages of the treatment options** | 2.4 (1.8) | 2.1 (1.6) | 0.496 | >0.999 |
| **5. My doctor helped me understand all the information** | 3.5 (1.4) | 2.6 (1.6) | 0.033 | 0.297 |
| **6. My doctor asked me which treatment option I prefer** | 3.6 (1.5) | 2.9 (1.7) | 0.119 | 0.714 |
| **7.My doctor and I thoroughly weighed the different treatment options** | 3.0 (1.6) | 2.5 (1.7) | 0.246 | 0.984 |
| **8. My doctor and I selected a treatment option together** | 3.9 (1.4) | 3.3 (1.7) | 0.133 | 0.714 |
| **9. My doctor and I reached an agreement on how to proceed** | 4.2 (1.1) | 3.6 (1.5) | 0.096 | 0.672 |

Individual items are scored from 0 to 5 on a six-point Likert scale ranging from 0 (″*completely disagree*″) to 5 (″*completely agree*″). The composite raw score was multiplied by twenty and divided by nine to obtain a new composite score that ranged from 0 to 100 where 100 reflected the highest possible level of shared decision-making.

^a^ Refers to the observed p-value before adjustment

^b^ Refers to the adjusted p-value using Holm-Bonferroni Method

Table 5. Comparison of SDMQ-Doc scores among the physicians in the intervention and control groups

|  |  | **Intervention** | **Control** |  | **^b^ Adjusted p-value (Holm-Bonferroni Method)** |
| --- | --- | --- | --- | --- | --- |
|  |  | **(N=30)** | **(n=30)** | **^a^p-value** |  |
| **Total SDMQ-Doc score, Mean (SD)** |  | 78.1 (14.1) | 73.2 (19.8) | 0.268 | >0.999 |
| **1.I made clear to my patient that a decision needs to be made** |  | 4.1 (0.7) | 3.9 (0.9) | 0.27 | >0.999 |
| **2.I wanted to know exactly from my patient how he/she wants to be involved in making the decision** |  | 3.8 (0.9) | 3.4 (1.2) | 0.153 | >0.999 |
| **3.I told my patient that there are different options for treating his/her medical condition** |  | 4.0 (1) | 3.9 (1.1) | 0.715 | >0.999 |
| **4.I precisely explained the advantages and disadvantages of the treatment options to my patient** |  | 3.7 (1) | 3.4 (1.3) | 0.24 | >0.999 |
| **5.I helped my patient understand all the information** |  | 3.8 (0.9) | 3.6 (1.3) | 0.555 | >0.999 |
| **6.I asked my patient which treatment option he/she prefers** |  | 4.0 (1.1) | 3.9 (1.2) | 0.661 | >0.999 |
| **7.My patient and I thoroughly weighed the different treatment options** |  | 3.6 (0.9) | 3.3 (1.3) | 0.286 | >0.999 |
| **8.My patient and I selected a treatment option together** |  | 3.9 (1) | 3.6 (1.2) | 0.255 | >0.999 |
| **9.My patient and I reached an agreement on how to proceed** |  | 4.1 (0.7) | 3.9 (0.8) | 0.311 | >0.999 |

Individual items are scored from 0 to 5 on a six-point Likert scale ranging from 0 (″*completely disagree*″) to 5 (″*completely agree*″). The total raw score was multiplied by twenty and divided by nine to obtain a new composite score that ranged from 0 to 100 where 100 reflected the highest possible level of shared decision-making.

^a^ Refers to the observed p-value before adjustment

^b^ Refers to the adjusted p-value using Holm-Bonferroni Method
